# Single-port-plus-one robot-assisted laparoscopic modified Lich-Gregoir direct nipple ureteral extravesical reimplantation in children with a primary obstructive megaureter

**DOI:** 10.3389/fped.2023.1238918

**Published:** 2023-11-02

**Authors:** Yufeng He, Shan Lin, Xinru Xu, Shaohua He, Huihuang Xu, Guangxu You, Jianglong Chen, Di Xu

**Affiliations:** ^1^ Department of Pediatric Surgery, Children Medical Center, Fujian Provincial Hospital, Fuzhou, China; ^2^ Shengli Clinical Medical College of Fujian Medical University, Fuzhou, China

**Keywords:** single-port-plus-one, robotic laparoscopic surgery, primary obstructive megaureter, direct nipple, ureteral extravesical reimplantation

## Abstract

**Purpose:**

To introduce a new technique of single-port-plus-one robotic laparoscopic-modified Lich-Gregoir direct nipple ureteral extravesical reimplantation and ascertain its validity in the treatment of pediatric primary obstructive megaureter.

**Methods:**

Between January 2021 and November 2021, we retrospectively analyzed the clinical data of 12 children with primary obstructive megaureter who were admitted to the Department of Pediatric Surgery of Fujian Provincial Hospital. All 12 children were treated with single-port-plus-one robotic laparoscopic Lich-Gregoir direct nipple ureteral extravesical reimplantation. Five of them were female and seven were male, including nine cases were simple obstructive type, while the remaining three cases were obstructive with reflux type. The mean age of the children was 17.33 ± 6.99 (10–36) months and the mean follow-up time was 14.16 ± 1.75 (12–17) months. Changes in preoperative and first-year postoperative parameters were compared**.**

**Results:**

The mean operative time for all 12 children was 123.58 ± 10.85 (110–145) min, with a mean internal operative time of 101.42 ± 0.85 (90–120) min, a mean operative bleeding time of 2.42 ± 0.67 (2–4) ml, and a mean hematuria duration of 16.08 ± 1.44 (14–19) h. The mean indwelling catheterization time was 2.25 ± 0.45 (2–3) days and the mean hospitalization time was 3.83 ± 0.39 (3–4) days. At the postoperative first year, the ureteral diameter, calyceal diameter, and anterior–posterior renal pelvic diameter were found to be significantly smaller than at the preoperative period (18.83 ± 3.21 mm vs. 6.83 ± 1.27 mm, 13.99 ± 3.58 mm vs. 3.5 ± 2.90 mm, and 34.92 ± 4.25 mm vs. 10.08 ± 1.88 mm, *P**** ***< 0.001). There was a significant increase in renal cortical thickness and the percentage of differential renal function (3.63 ± 1.66 mm vs. 5.67 ± 1.88 mm, 33.75 ± 2.77 mm vs. 37.50 ± 1.31 mm, *P* < 0.001). The resolution rate of obstruction was 100% and no child developed DeNovo vesicoureteral reflux.

**Conclusion:**

The technique of modified Lich-Gregoir direct nipple ureteral extravesical reimplantation can help maintain the physiological direction of the ureter and at the same time enhance the effectiveness of antirefluxing in robotic surgery. The design of a single-port-plus-one wound can produce a cosmetic appearance by concentrating and hiding the wound around the umbilicus. This modified reimplantation procedure has the potential to become a promising technique in the robot-assisted treatment of primary obstructive megaureter.

## Introduction

The term Primary Obstructive Megaureter (POM), which was coined by Caulk in 1923 (1), results in hindered urine evacuation and dilates the ureter and renal collecting system (2). Most will resolve over time. However, in approximately 20% of patients with uncontrolled urinary tract infection (UTI) or high-grade or progressive obstruction, ureteral reimplantation is required (3). Traditionally, open ureteral reimplantation is the gold standard for primary obstructive megaureter. In the past decade, pediatric urologists have frequently implemented minimally invasive procedures by using laparoscopic, robotic-assisted, and other devices. The Da Vinci robot–assisted laparoscopic surgery platform, which is characterized by a 6° freedom articulation, tremor filtering, and stereoscopic vision, provides distinct advantages in intracorporeal reparation and suturing (4). These advantages have expanded the role of robot-assisted surgery in complex lower urinary tract reconstructive surgeries, which are possibly time-consuming due to the narrow pelvic space (5). To promote the use of robot-assisted surgery for the treatment of obstructed megaureter and to achieve optimal outcomes, herein, we implemented a novel technique called “single-port-plus-one robotic laparoscopic-modified direct nipple Lich-Gregoir extravesical reimplantation”, in an attempt to minimize technical difficulties. We reviewed our prospectively collected data and assessed the short-term safety and feasibility of the procedure.

## Patients and methods

Between January 2021 and November 2021, we retrospectively reviewed 12 patients with POM who were admitted to the Department of Pediatric Surgery of Fujian Provincial Hospital, of whom five were females and seven were males. Their mean age was 17.33 ± 6.99 (10–36) months and the mean follow-up time was 14.16 ± 1.75 (12–17) months. Out of 12 patients, five are on the right side and seven are on the left side. The patients on the right are all obstructive. Among the patients on the left, four are obstructive and three are obstructive with reflux. A prenatal diagnosis was carried out in all 12 patients. Two patients experienced abdominal pain and three presented with UTIs.

All patients underwent a preoperative magnetic resonance urography (MRU), urological ultrasound, diuretic renogram (99 m Tc-DTPA), renal static imaging (99 m Tc-DMSA), and voiding cystourethrography (VCUG). All patients presented with a worsening dilatation (6) of 5 UTDP3 grade and 7 UTDP2 grade, and all of them had a deteriorating differential renal function (DRF) and obstructive curves on serial scans. A preoperative VCUG was done in all patients, and three of them had a vesicoureteral reflux (VUR), with grade 1 in two and grade 3 in one.

All patients underwent single-port-plus-one robotic laparoscopic-modified direct nipple Lich-Gregoir extravesical reimplantation. Five of them underwent ureteral tapering repair, which was performed by using Hendren's technique. Informed consent was obtained from the parents of the children, and the ethical review of the study was performed in our institution (ethics approval number: K2020-12-033).

In the postoperative second month, the patients were readmitted to the hospital for cystoscopy, observation of the ureteral orifice pattern, and removal of the double J tube. On the first year after surgery, the patients again returned for a urological ultrasound, MRU, diuretic nephrography, renal static imaging (99m Tc-DMSA), and VCUG to assess their recovery. Also, on the first year after surgery, all patients underwent a cystoscopy for an observation of the ureteral orifice, and the F3 ureter catheter was passed through the ureteral orifice to confirm no kicking in the ureter.

### Inclusion and exclusion criteria

**Inclusion criteria**: POM patients with symptoms such as febrile UTIs or pain were included in the study (7). Asymptomatic patients with a DRF below 40% and which was associated with massive or progressive hydronephrosis, or a drop of >5% in differential function, were included.

**Exclusion criteria**: Patients with secondary giant ureters caused by ectopic ureteral opening, neurogenic bladder, posterior urethral valve, or urethral stenosis were excluded from the study (7).

### Surgical procedures

All robot-assisted laparoscopic surgeries were performed by the same surgeon and surgical team.

After administering a successful general anesthesia, the patients were kept in the supine position, with head low and foot high, the bilateral upper limbs were placed in a “surrender” position, and the bilateral upper limbs were slightly opened. All parts of the body under pressure were padded with a sponge and fixed with bandages. Disinfecting, draping, and urethral catheterization were performed before the starting the single-port-plus-one robotic laparoscopic-modified Lich-Gregoir direct nipple ureteral extravesical reimplantation.

**Figure 1:**
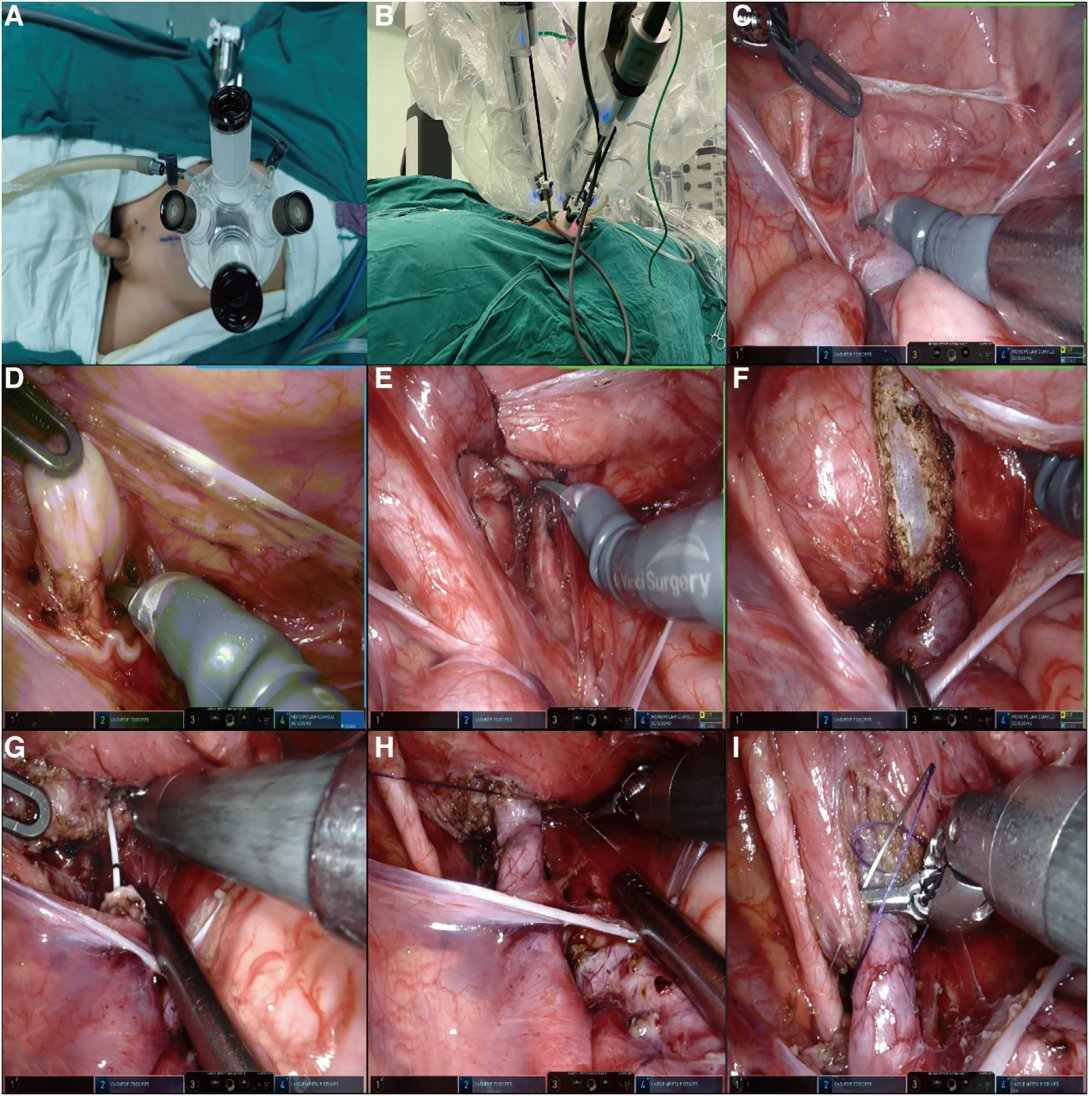
(**A**,**B**) A 2.5–3 cm curved incision was made along the edge of the umbilical cord to place a quadruple-channel puncture device. Another 8 mm incision was made 6 cm away from 3D camera port III on the right side of the abdomen for inserting the robotic operating port II. (**C**,**D**) We established a “peritoneal window,” in which the vas or uterine artery was directly identified from the ureter. (**E**,**F**) The direction of the detrusor tunnel was designed obliquely. The bladder detrusor was cut and disassembled until bulging of the bladder mucosa occurred, the length of the incision is five times the normal width of the ureter. (**G**,**H**) The overdilated ureter was cut and shaped and a double J tube was inserted into the ureter. The 1.5 cm end of the ureter was placed into the bladder. The seromuscular layer of the ureter and the bladder mucosa were sutured at 3, 6, 9, and 12 o' clock positions with 6-0 PDS. Finally, four stitches were made in each quadrant to complete the anastomosis. (**I**) A “down–top” method was applied to complete an intermittent incision of the cut detrusor suture with 4-0 Vicryl.

Although the da Vinci system Xi has four robotic arms, we used only three [three-dimensional (3D) camera arm III, operating arm II, and operating arm IV] and inserted a quadruple-channel puncture device to improve cosmetic appearance. We made a 2.5–3 cm curved incision along the edge of the umbilical cord to place a quadruple-channel puncture device, the four channels of which were used to insert an 8 mm 3D camera port III and an 8 mm operating port IV and were also used as assistant channels in a subsequent operation. Artificial pneumoperitoneum was established with a pressure rate of 10 mmH_2_O and a flow rate of 4 L/min. Another 8 mm incision was made 6 cm away from 3D camera port III on the right side of the abdomen for inserting robotic operating port II, as shown in Figures 1A,B.

Instead of using the traditional two peritoneal incisions to identify the ureter, we devised a new method of creating a “peritoneal window.” We used a transverse incision to open the peritoneal layer along the surface of the posterior bladder wall, dissected the peritoneal layer from the posterior bladder wall on both sides, and then used an undamaged vessel clip to fix the peritoneal layer with the surrounding tissue. In these ways, we established a “peritoneal window,” in which the vas or uterine artery could be directly identified from the ureter, as shown in Figures 1C,D.

The direction of the detrusor tunnel was designed obliquely and a local electrocoagulation marker was generated. Traction sutures of the posterior bladder wall were inserted through the abdominal wall above the middle of the pubic symphysis and pulled out from the inserted position. In this way, the posterior bladder wall was pulled obliquely to the abdominal wall, and the detrusor tunnel was kept straight, which was convenient for cutting and dissecting. Inject saline into the bladder through a catheter to fill it. The bladder detrusor was cut and disassembled until bulging of the bladder mucosa occurred, the length of the incision is five times the normal width of the ureter. The outward and upward detrusor tunnel was more in line with the physiological direction of the ureter, as shown in Figures 1E,F.

The stricture segment of the ureter was resected, the overdilated ureter was cut and shaped as needed, and a double J tube was inserted into the ureter. Then, the 1.5 cm end of the ureter was placed into the bladder. Next, we sutured the seromuscular layer of the ureter and the bladder mucosa at 3, 6, 9, and 12 o’ clock positions with 6-0 PDS, and finally, four stitches were made in each quadrant to complete the anastomosis, as shown in Figures 1G,H.

The “down–top” method was applied to complete an intermittent incision of the cut detrusor suture with 4-0 Vicryl, as shown in Figure 1I. Finally, the peritoneal layer of the posterior bladder wall was closed and the wound was sutured.

## Results

The mean operative time for all 12 children was 123.58 ± 10.85 (110–145) min, with a mean internal operative time of 101.42 ± 0.85 (90–120) min, a mean operative bleeding of 2.42 ± 0.67 (2–4) ml, and a mean hematuria duration of 16.08 ± 1.44 (14–19) h. The mean indwelling catheterization time was 2.25 ± 0.45 (2–3) days, and the mean hospitalization time was 3.83 ± 0.39 (3–4) days, as shown in Table 1.

**Table 1:** Basic patient information on the modified Lich surgery.

NO	Sex	Age (months)	Side	The type of POM^a^	UTD grade	Operation time (mins)	Blood loss (ml)	Gross hematuria (h)	Indwelling catheterization time (days)	Hospitalization (days)	Complication
1	Boy	13	L	O	P3	112	2	15	2	4	
2	Girl	10	R	O	P2	130	3	16	2	4	
3	Boy	12	L	O	P3	145	3	18	2	4	
4	Boy	15	L	O	P3	123	2	19	2	4	
5	Girl	17	L	OR	P3	116	2	17	2	4	
6	Boy	24	R	O	P2	117	2	15	2	3	
7	Girl	36	L	OR	P3	135	2	16	3	4	
8	Boy	20	L	O	P2	134	4	15	3	3	
9	Girl	18	R	O	P2	110	3	14	3	4	
10	Boy	16	L	OR	P2	125	2	17	2	4	
11	Boy	13	R	O	P2	124	2	16	2	4	
12	Girl	14	R	O	P2	112	2	15	2	4	

UTD, urinary tract dilation.

According to the system of the American Pediatric Association for classifying the megaureter, O represents obstruction without refluxing, and QR represents obstruction with refluxing.

^a^ Represents the source of POM typing.

**Figure 2:**
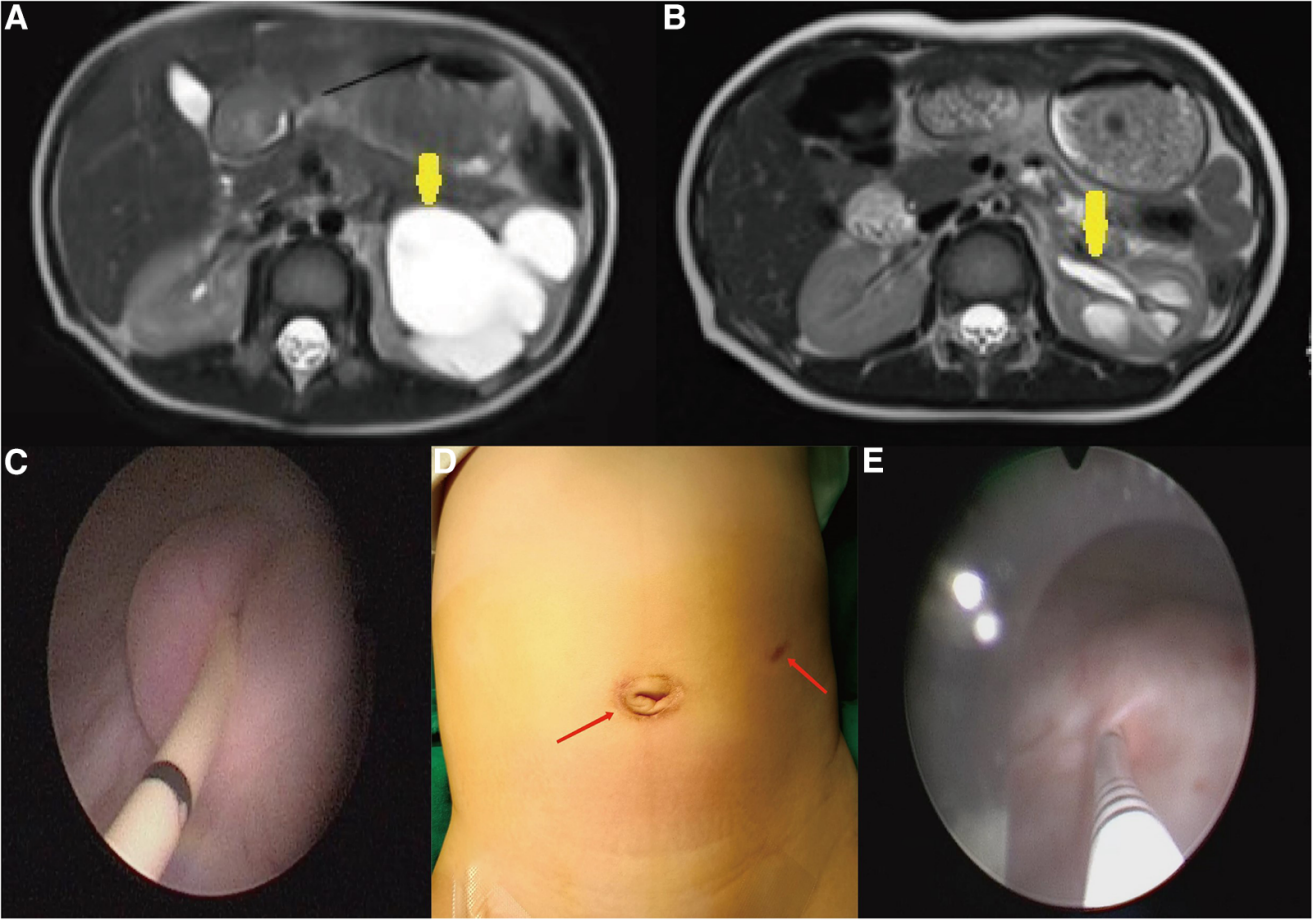
(**A**,**B**) At the postoperative first year, there was a significant reduction in APRPD and ureteral diameter compared with those before the operation (yellow arrow). (**C**) The end of the ureter that was placed into the bladder was found to turn into nipples automatically in cystoscopy at the postoperative second month. (**D**) Cosmetic appearance of the wound at the postoperative second month (the scar is marked by a red arrow). (**E**) In the first year after surgery, the F3 ureter catheter was passed through the ureteral orifice smoothly into the ureter, confirming no kicking in the ureter.

At the postoperative first year, the ureteral diameter, calyceal diameter, and anterior–posterior renal pelvic diameter (APRPD) were found to be significantly smaller than at the preoperative period (18.83 ± 3.21 mm vs. 6.83 ± 1.27 mm, 13.99 ± 3.58 mm vs. 3.5 ± 2.90 mm, and 34.92 ± 4.25 mm vs. 10.08 ± 1.88 mm, *P* < 0.001) (Figures 2A,B: the yellow arrow). There was a significant increase in renal cortical thickness and the DRF percentage (3.63 ± 1.66 mm vs. 5.67 ± 1.88 mm, 33.75 ± 2.77 mm vs. 37.50 ± 1.31 mm, *P* < 0.001), as shown in Table 2.

**Table 2:** Comparison of the preoperative parameters and 1-year postoperative parameters.

	Ureteral diameter (mm), mean ± SD	Calyceal diameter (mm), mean ± SD	APRPD (mm), mean ± SD (mm)	Renal cortex thickness (mm)	DRF %, mean ± SD
Preoperative	18.83 ± 3.21	13.99 ± 3.58	34.92 ± 4.25	3.63 ± 1.66	33.75 ± 2.77
First-year postoperative	6.83 ± 1.27	3.5 ± 2.90	10.08 ± 1.88	5.67 ± 1.88	37.50 ± 1.31
*d*	12.00 ± 2.52	10.49 ± 4.20	24.83 ± 3.19	2.14 ± 0.60	3.75 ± 1.71
*t*	12.30	8.638	18.501	−12.168	−4.24
*P*	<0.001	<0.001	<0.001	<0.001	<0.001

In the postoperative period, no patient developed urinary retention, urinary extravasation, and wound infection. Two patients developed postoperative UTI treated conservatively and no one demonstrated reflux on VCUG. The resolution rate of obstruction was 100% and no patient developed DeNovo VUR.

**Figure 3:**
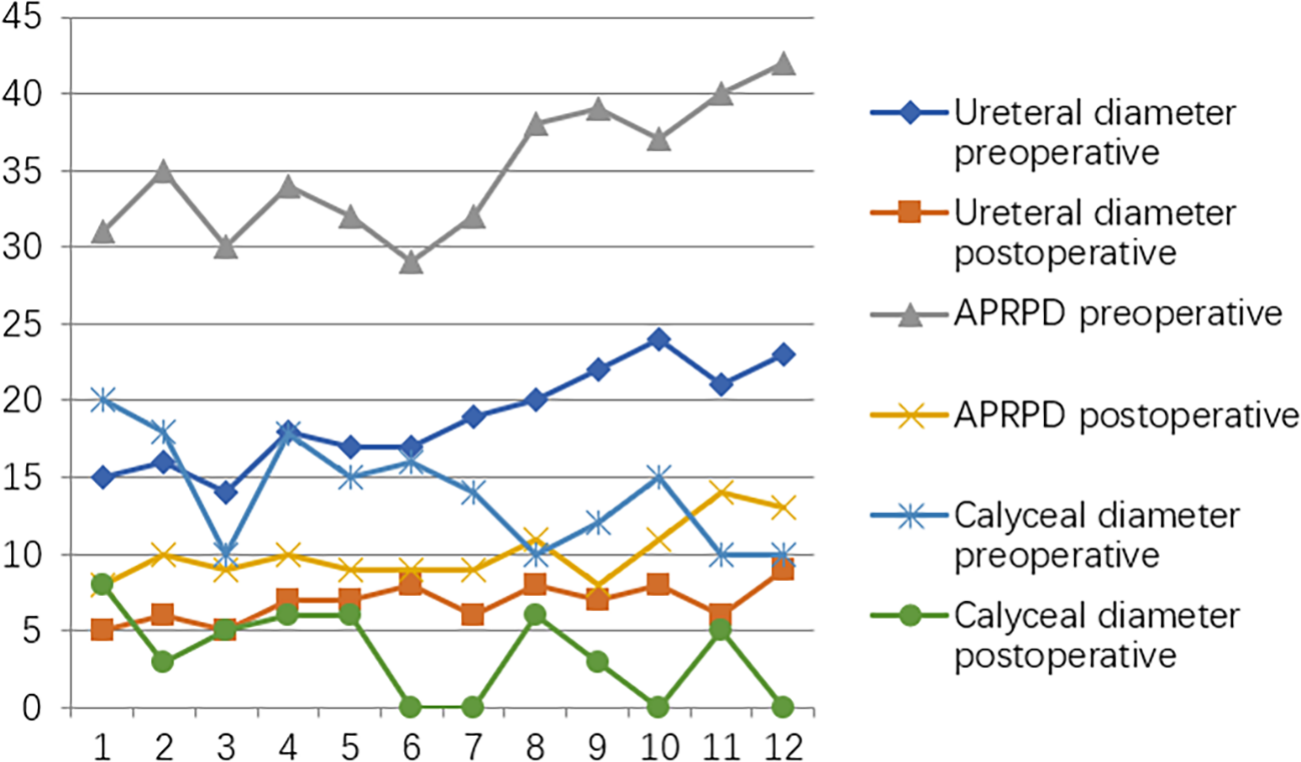
Trends in variations in ureteral diameter, APRPD, and calyceal diameter at the preoperative and one-year postoperative time points.

**Figure 4:**
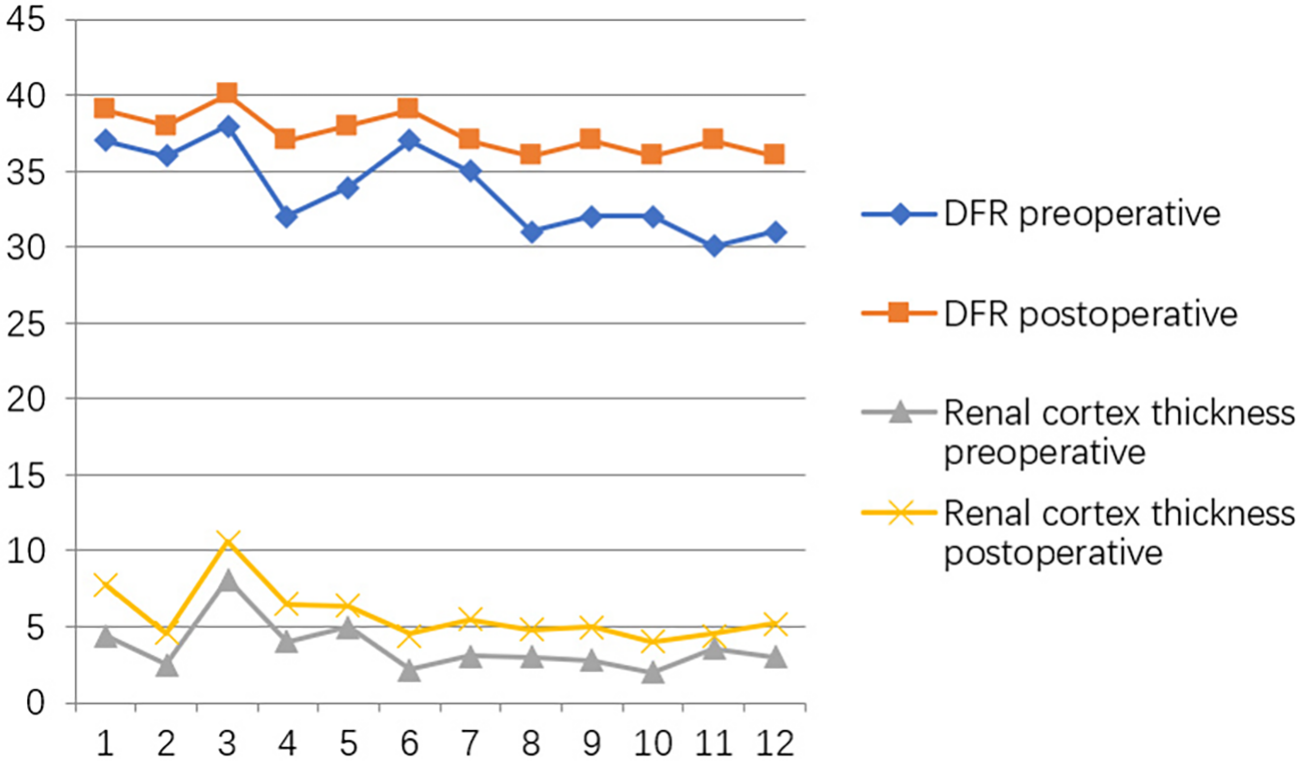
Trends in variations in renal cortex thickness and DRF variation at the preoperative and one-year postoperative time points.

At the postoperative second month, the patients were readmitted to the hospital for a cystoscopy for removal of the double J catheter, and the end of the ureter that was placed into the bladder was found to turn into nipples automatically, as shown in Figure 2C, and the wounds were cosmetic, as shown in Figure 2D. On the first year after surgery, all patients underwent cystoscopy for observation of the ureteral orifice again, and the F3 ureter catheter was passed through the ureteral orifice smoothly into the ureter, which confirmed no kicking in the ureter, as shown in Figure 2E. The trends of variations in the ureteral diameter, APRPD, and calyceal diameter at the preoperative and 1-year postoperative time points are shown in Figure 3. The trends of renal cortex thickness and DRF variation at the preoperative and 1-year postoperative time points are shown in Figure 4.

## Discussion

According to the International Classification of Diseases, congenital megaureter can be classified either as obstructed and refluxing or as unobstructed and unrefluxing (7). The treatment goal of POM is to relieve obstruction while establishing a new and effective antireflux mechanism. The primary minimally invasive surgical approaches for the correction of an obstructed megaureter that were used so far and are still used today are laparoscopic extravesical reimplantation and the pneumovesical laparoscopic approach (8, 9). Pneumovesical laparoscopic approaches such as Cohen and Leadbetter are often technically challenging and require a long curve, even for the most experienced laparoscopic surgeons. In infants and fat adolescents, they are often more challenging due to the small bladder volume and thickness of the abdominal wall (10, 11). In recent years, the robot-assisted laparoscopic platform with a stereoscopic vision and flexible arms has provided a new opportunity for the laparoscopic extravesical approach for POM.

Two robotic-assisted laparoscopic ureteral extravesical reimplantation methods for the treatment of POM have been described in recent studies. One is robotic-assisted extravesical cross-trigonal ureteral reimplantation (12, 13). In 2020 (13), Neheman et al. described a novel surgical approach in the pediatric population and found that anastomosis was more ergonomic when performing a horizontal detrusorrhaphy, as opposed to a vertical one, and the submucosal tunnel was easier to extend. But the technique also had disadvantages in the form of challenges with endoscopic ureteral intubation during follow-up, if needed, postoperative cytoplasm, and urinary retention.

The other method is robotic-assisted extravesical Lich-Gregoir ureteral reimplantation, which is mainly used in high-grade VUR surgeries (14, 15). We reviewed the currently published literature on robot-assisted laparoscopic ureteral reimplantation (RALUR) and found that the VUR resolution rates widely ranged from 66.7% to 100%, which may be attributed to a submucosal tunnel of insufficient length (15).

In this investigate, we implemented a new method by maintaining the vertical submucosal tunnel in the Lich-Gregoir technique and adding ureteral direct nipple implantation to produce an antireflux effect. As indicated by previous reports, traditional ureteral nipple implantation is not convenient to perform in adults (16, 17). Al-Shukri and Alwan (18) first reported direct nipple ureteroneocystostomy in adults with a ureteral stricture (19). In 2014, Fu et al. also used this method in robot surgeries in adult megaureters. In our new technique, we used this method in the pediatric population. In all 12 children in our study, the new ureteral orifice automatically acquired a papillary shape upon cystoscopy 2 months after surgery. At the postoperative first year, the ureteral diameter, calyceal diameter, and APRPD were found to be significantly smaller than at the preoperative period. There was a significant increase in renal cortical thickness and DRF percentage. The resolution rate of obstruction was 100% and no child developed DeNovo VUR on VCUG. The results of the postoperative tests showed that this strategy was simple, safe, and feasible. In particular, the ureteral direct nipple implantation also shortened the anastomosis time, and the vertical submucosal tunnel remained in the physiological direction of the ureter, which was confirmed by the smooth passage of the 3F ureteral catheter upon cystoscopy at the postoperative 1-year period.

According to research by Leissner et al. of human cadaver dissection, the main portion of the pelvic plexus is located at about 1.5 cm dorsal and medial to the ureterovesical junction. The bundles of the pelvic plexus end at the distal ureter, trigone, and rectum (20). The oblique detrusor tunnel designed in the Lich-Gregoir technique may help preserve the neurovascular bundles and avoid potential voiding issues such as urinary retention during mobilization of the ureter.

Traumas experienced by children will get magnified as they grow up. That is why minimally invasive surgery has gradually become the mainstream treatment in the pediatric population in recent years. Unlike the four scattered wounds (three robotic port wounds and one assisted laparoscopic port wound) in the abdomen wall (21, 22), the wounds created by the single-site-plus-one technique are concentrated and hidden around the umbilical cord, which are more concealed when children grow up. When 5 mm robotic arms or the Da Vinci SP system are used by Chinese surgeons in the future, there is a possibility that all robotic arms are concentrated in a single-site port to realize a successful single-site robot-assisted surgery.

## Conclusion

The technique of modified Lich-Gregoir ureteral direct nipple extravesical reimplantation can help maintain the physiological direction of the ureter and at the same time enhance the effectiveness of antirefluxing in robotic surgery. The design of a single-port-plus-one wound can produce a cosmetic appearance by concentrating and hiding the wound around the umbilicus. This modified reimplantation procedure has the potential to become a promising surgical technique in the robot-assisted treatment of POM.

## Data Availability

The raw data supporting the conclusions of this article will be made available by the authors, without undue reservation.
